# Retrospection of Seldom-Known Causes and Presentations of Partial Empty Sella Syndrome

**DOI:** 10.7759/cureus.44494

**Published:** 2023-08-31

**Authors:** Kushagra Khanna, Rajika Khanna, Sunil Kumar

**Affiliations:** 1 Department of Medicine, Jawaharlal Nehru Medical College, Datta Meghe Institute of Medical Sciences, Wardha, IND; 2 Department of Medicine, Kasturba Medical College, Manipal Academy of Higher Education, Udupi, IND

**Keywords:** bradycardia, hypotensive crisis, common mimickers of old age, elderly population, partial empty sella syndrome

## Abstract

Pituitary gland shrinkage or flattening obscures it from view on an MRI, giving the impression that it is an empty sella. On the other hand, if some viable pituitary gland tissue is still seen on the MRI scan, a diagnosis of partial empty sella can be made. Diminished physiological and functional reserve in the elderly can result in a bad prognosis if not treated early. Therefore, there is a need to become familiar with fewer known causes and presentations of the empty sella or partial empty sella syndrome in older patients. We report a case of a 71-year-old female with multiple known ailments presenting with hypotension as the sole symptom. It urged us to investigate further and reach the root cause as partial empty sella syndrome with panhypopituitarism.

## Introduction

The primary empty sella (PES) is characterized by several diseases, such as a congenitally deficient sellar diaphragm formation, suprasellar causes including a steady or sporadic increase in intracranial pressure, and volumetric alterations in the pituitary (as seen during pregnancy) [1,2]. Additionally, pituitary adenomas that experience spontaneous necrosis (ischemia or bleeding) might result in secondary empty sella (SES). Other well-known reasons include autoimmune, infections, radiation, traumatic, specific medications, and surgical procedures. This syndrome, which is clinically asymptomatic in 50% of cases and in many cases is an incidental radiological finding [3], may cause severe endocrine abnormalities, necessitating further investigation and the need to perform pituitary function tests if found on an MRI performed based on clinical suspicion or during scans for other purposes.

## Case presentation

**Figure 1:**
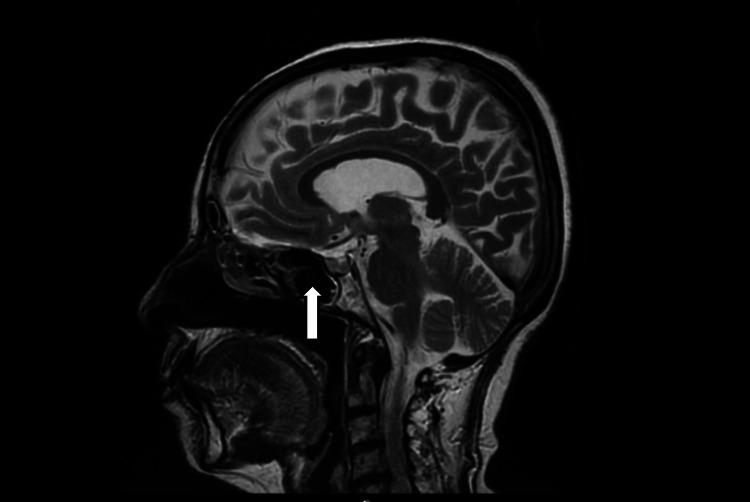
T2-weighted MRI in the mid-sagittal section that demonstrates a fluid-filled sella turcica with a flattened pituitary that is just visible at the base of the pituitary fossa

**Figure 2:**
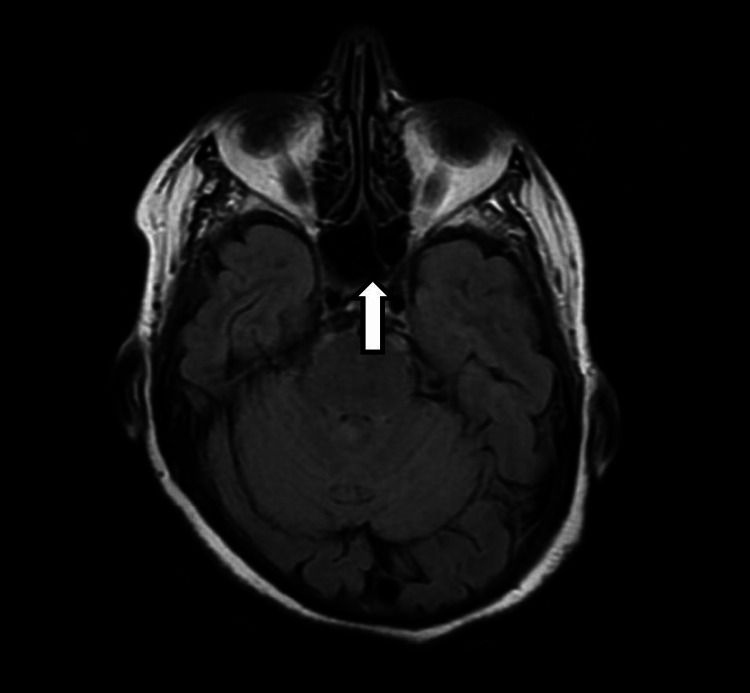
A T1-weighted coronal section that illustrates the enlarged, fluid-filled sella

**Figure 3:**
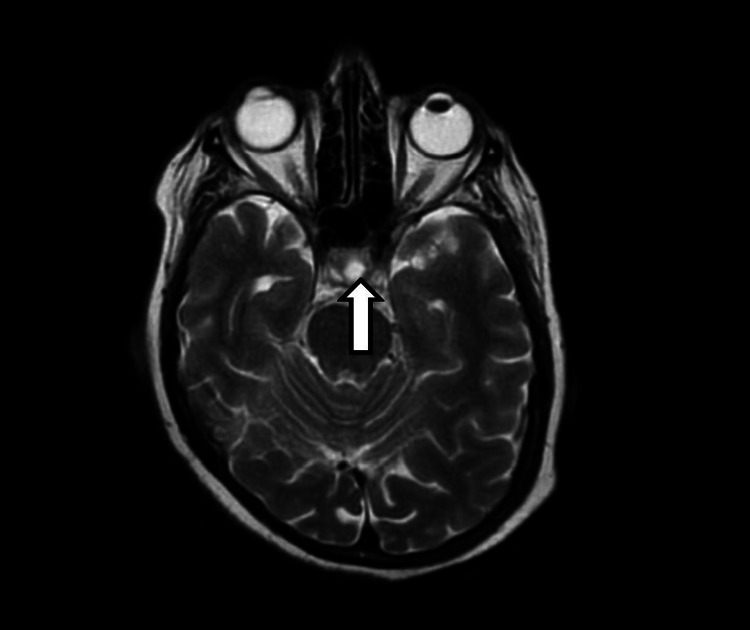
T2-weighted (fluid-attenuated) coronal section that illustrates the increased signal intensity of the CSF occupying the sella turcica

A 71-year-old female with a known case of hypertension, type II diabetes mellitus, hypothyroidism, and left breast cancer on hormonal therapy came to the hospital complaining of fatigue and loss of appetite. She felt nauseous but had no episodes of vomiting. The patient has been experiencing myalgia for the past three to four days and had a weight loss of 2 kg in two months. On arrival, the BP recorded was 88/60 mmHg in the hospital. Other vitals general and systemic examinations were unremarkable. Her labs showed reduced cortisol levels. Adrenocorticotropic hormone (ACTH), thyroid-stimulating hormone (TSH), and follicle-stimulating hormone (FSH) are also low (Table 1). However, prolactin was in the normal range. A cosyntropin test was done, and it was suggestive of secondary adrenal insufficiency. An MRI of the brain showed partial empty sella syndrome (Figures 1-3). A dual x-ray absorptiometry scan was done, and it was suggestive of bone osteopenia. She started bisphosphonates and calcium supplements. Her blood sugars were slightly deranged. She was started on oral hypoglycemic agents. A medical oncology opinion was sought. It was planned to continue hormonal therapy with letrozole. Her general condition improved; hence, she was discharged on oral steroids. An endocrinology consult was sought. She was started on a steroid supplement. Her BP was monitored closely, and it started to improve.

**Table 1:** Investigations WBC: white blood cell, TSH: thyroid-stimulating hormone, T3: triiodothyronine, T4: thyroxine, ACTH: adrenocorticotropic hormone, FSH: follicle-stimulating hormone

Investigations	Result	Reference range
Hemoglobin	12.3 g/dL	12.0-15.0 g/dL
Platelet count	200.0 x 10³/µL	150-400 x 10³/µL
Total WBC	5.7 x 10³/µL	4.0-10.0 x 10³/µL
Neutrophils	32.4%	42-74%
Lymphocytes	41.3%	18-44%
Monocytes	15.6%	5.0-13.0%
Eosinophils	9.7%	1.0-8.0%
Sodium (serum)	129.0 mmol/L	136-145 mmol/L
Total protein (serum)	6.00 g/dL	6.4-8.3 g/dL
Albumin (serum)	3.20 g/dL	3.5-5.2 g/dL
TSH (serum)	0.020 µIU/mL	0.27-4.20 µIU/mL
Free T3 (serum)	4.23 pg/mL	2.0-4.4 pg/mL
Free T4 (serum)	1.00 ng/dL	0.93-1.7 ng/dL
Cortisol (serum)	0.430 µg/dL	0800 hr-4.82-19.5 µg/dL
ACTH (serum)	<1.5 pg/ml	7.2-63.3 pg/mL
FSH (serum)	2.2 mIU/mL	Males: 1.5 -12.4 mIU/mL, female: non-pregnant mid-follicular phase, 3.5-12.5 mIU/mL, mid-cycle, 4.7-21.50 mIU/mL, mid-luteal, 1.7-7.7 mIU/mL, post-menopausal, 25.8-134.8 mIU/mL
Calcium (serum)	8.4 mg/dL	8.8-10.2 mg/dL
Vitamin D (serum)	23.80 ng/ml	<20 ng/ml Deficient, 21-29 ng/ml Insufficient, >30 ng/ml normal

## Discussion

Lower limb edema, hyponatremia, adrenal insufficiency, and hypotension are common conditions that the elderly can present with individually. To detect a partially empty sella, these can constitute exclusion symptoms. In addition to these, there may be a few more manifestations, which are covered in more detail below, which could point to the diagnosis. Hence, it is essential to take into account the syndrome's less common etiologies as well.

The causes of complex or refractory hyponatremia requiring endocrinology consultation were investigated in a case series of a sample size of 185 individuals over a 20-year follow-up period [4]. According to the study, only 12 (6%) had empty sella, hypervolemia, or hyponatremia with diuresis, while 28 (15%) had secondary adrenal insufficiency as the primary cause of their hyponatremia. This finding was cited in a case report [5], where the failure to resolve painful leg swelling in a case initially diagnosed as hypervolemic hyponatremia with diuresis prompted them to conduct additional investigations. They reiterated the significance of using a strategic approach in evaluating hyponatremia to find relatively rare yet easily treatable underlying causes. Adrenal insufficiency is typically seen in patients with complete rather than partial empty sella. However, in our patient, who had a partially empty sella, hypocortisolism was sufficient to produce severe hyponatremia, which might have had life-threatening repercussions if undiagnosed. This may be accounted for by the lack of physiological cortisol regulation in ADH, which leads to hypervolemic hyponatremia.

A case of a 75-year-old male with hypopituitarism masquerading as failure to thrive has been drafted [6]. They highlighted that hypopituitarism's symptoms could be vague and resemble aging-related changes. However, several symptoms, such as pallor, postural hypotension, urine incontinence, falls, lethargy, confusion, and flexion contractures, should alert doctors to an unconventional diagnosis of hypopituitarism in the elderly. They described hypoglycemia and hyponatremia as common sequelae of hypopituitarism and should not be dismissed downright as failure to thrive or frailty in the elderly.

A case of empty sella syndrome was discovered during postoperative hypotension and respiratory failure [7]. They thought that the patient's procedure had caused a pituitary crisis. This example emphasizes the significance of considering pituitary hormone insufficiency when a perioperative respiratory and hemodynamic failure occurs. They strongly recommend including a thyroid function test in the preoperative evaluation, which could be a guide to rule out linked diseases if there is any irregularity.

A 66-year-old male was found to have bradycardia pre-operatively when hospitalized for an elective transforaminal lumbar interbody fusion at the level of L5-S1, as discussed in a case study [8]. After additional workup, he was found to have low levels of free thyroxine (FT4) and TSH. He had a history of hypotestosteronemia as well. At the time of admission, he had a diagnosis of central hypothyroidism. The partly empty sella syndrome diagnosis was made after a brain MRI scan using MRI. They emphasized that empty sella syndrome can manifest as bradycardia and that clinicians must consider a broad differential that includes alternative non-cardiac explanations for typical cardiac symptoms such as bradycardia.

Pituitary apoplexy that was temporally related to systemic chemotherapy delivery has been reviewed [9]. One week after beginning chemotherapy with bleomycin, etoposide, and cisplatin, a 31-year-old male with newly diagnosed metastatic testicular cancer had headaches, nausea, and a deficiency in his right side of the visual field. He had immediate transnasal excision after CT, and MRI revealed a hemorrhage within a pituitary macroadenoma consistent with pituitary apoplexy, revealing yet another startling source of pituitary failure.

GnRH agonists are generally safe, but some patients, particularly those with a pituitary adenoma, may experience pituitary apoplexy [10]. This complication should be known to doctors because it may be fatal. Hence, pituitary apoplexy patients should be treated using a multidisciplinary team approach.

A 73-year-old male scheduled for surgery on a colorectal malignancy exhibiting clinical and biochemical signs of acromegaly and an empty sella on pituitary MRI was the subject of a case study by Bestepe et al. [11]. They explained that it is yet unknown what causes acromegaly and empty sella to be related. Pituitary adenoma apoplexy can lead to the production of necrosis and, ultimately, empty sella. We considered PES because our patient had no prior history of pituitary apoplexy, and we could not identify any causes for SES.

## Conclusions

Partial empty sella syndrome is a rare and incidental finding in the elderly. It is imperative to do a thorough investigation in case of clinical suspicion or after incidental radiological diagnosis of the syndrome to prevent grave consequences in untreated elderly patients.
